# Differential Mechanisms of Septic Human Pulmonary Microvascular Endothelial Cell Barrier Dysfunction Depending on the Presence of Neutrophils

**DOI:** 10.3389/fimmu.2018.01743

**Published:** 2018-08-02

**Authors:** Lefeng Wang, Sanjay Mehta, Yousuf Ahmed, Shelby Wallace, M. Cynthia Pape, Sean E. Gill

**Affiliations:** ^1^Centre for Critical Illness Research, Lawson Health Research Institute, London, ON, Canada; ^2^Department of Medicine, Western University, London, ON, Canada; ^3^Division of Respirology, Western University, London, ON, Canada; ^4^Department of Physiology and Pharmacology, Western University, London, ON, Canada

**Keywords:** human sepsis, endothelial barrier dysfunction, neutrophil, caspase activity, cell co-culture

## Abstract

Sepsis is characterized by injury of pulmonary microvascular endothelial cells (PMVEC) leading to barrier dysfunction. Multiple mechanisms promote septic PMVEC barrier dysfunction, including interaction with circulating leukocytes and PMVEC apoptotic death. Our previous work demonstrated a strong correlation between septic neutrophil (PMN)-dependent PMVEC apoptosis and pulmonary microvascular albumin leak in septic mice *in vivo*; however, this remains uncertain in human PMVEC. Thus, we hypothesize that human PMVEC apoptosis is required for loss of PMVEC barrier function under septic conditions *in vitro*. To assess this hypothesis, human PMVECs cultured alone or in coculture with PMN were stimulated with PBS or cytomix (equimolar interferon γ, tumor necrosis factor α, and interleukin 1β) in the absence or presence of a pan-caspase inhibitor, Q-VD, or specific caspase inhibitors. PMVEC barrier function was assessed by transendothelial electrical resistance (TEER), as well as fluoroisothiocyanate-labeled dextran and Evans blue-labeled albumin flux across PMVEC monolayers. PMVEC apoptosis was identified by (1) loss of cell membrane polarity (Annexin V), (2) caspase activation (FLICA), and (3) DNA fragmentation [terminal deoxynucleotidyl transferase dUTP nick end labeling (TUNEL)]. Septic stimulation of human PMVECs cultured alone resulted in loss of barrier function (decreased TEER and increased macromolecular flux) associated with increased apoptosis (increased Annexin V, FLICA, and TUNEL staining). In addition, treatment of septic PMVEC cultured alone with Q-VD decreased PMVEC apoptosis and prevented septic PMVEC barrier dysfunction. In septic PMN–PMVEC cocultures, there was greater trans-PMVEC macromolecular flux (both dextran and albumin) vs. PMVEC cultured alone. PMN presence also augmented septic PMVEC caspase activation (FLICA staining) vs. PMVEC cultured alone but did not affect septic PMVEC apoptosis. Importantly, pan-caspase inhibition (Q-VD treatment) completely attenuated septic PMN-dependent PMVEC barrier dysfunction. Moreover, inhibition of caspase 3, 8, or 9 in PMN–PMVEC cocultures also reduced septic PMVEC barrier dysfunction whereas inhibition of caspase 1 had no effect. Our data demonstrate that human PMVEC barrier dysfunction under septic conditions *in vitro* (cytomix stimulation) is clearly caspase-dependent, but the mechanism differs depending on the presence of PMN. In isolated PMVEC, apoptosis contributes to septic barrier dysfunction, whereas PMN presence enhances caspase-dependent septic PMVEC barrier dysfunction independently of PMVEC apoptosis.

## Introduction

Sepsis, defined as organ dysfunction due to a dysregulated host response to infection, is the most common cause of death in Intensive Care Units (ICU), and is one of the leading healthcare expenses for patients in the hospital consuming up to 45% of total ICU costs (1–3). Mortality in sepsis is due to multiple organ dysfunction, most commonly the lung leading to acute respiratory distress syndrome (ARDS), but also systemic organs such as heart, brain, and kidneys (1, 2, 4). Septic organ dysfunction is due to an overwhelming systemic inflammatory process, characterized by the enhanced production and release of a plethora of soluble inflammatory mediators, including bacterial-derived lipopolysaccharide (LPS) and various host-derived cytokines [e.g., tumor necrosis factor (TNF) α, interleukin (IL) 1β, interferon (IFN) γ], as well as the activation of both circulating [e.g., polymorphonuclear leukocytes (PMN)] and tissue-resident (e.g., macrophages) inflammatory cells (4–6).

Septic organ dysfunction is also recognized to be due, in large part, to significant activation and dysfunction of the microvasculature of individual organs. Microvascular dysfunction is characterized by impaired barrier function (increased permeability leading to extra-vascular leak of protein-rich edema) and PMN influx into organs (7–10), microvascular thrombosis (11), and impaired distribution of blood flow in microvascular beds (12). Moreover, microvascular dysfunction is clinically important, as it has been documented early in the course of sepsis in humans, and is associated with increased mortality (13, 14), especially if it persists over time (15).

The mechanism of microvascular dysfunction in sepsis is primarily thought to be activation and dysfunction of microvascular endothelial cells (MVEC). This septic activation and dysfunction of MVEC leads to severe tissue edema, inflammation, and ultimately, organ dysfunction and failure. Multiple factors promote septic MVEC activation and dysfunction, including cytokine signaling, mechanical interaction with activated leukocytes, and exposure to harmful leukocyte-derived molecules, such as proteases and oxidants [e.g., nitric oxide (NO)] (4, 8, 16–21). Moreover, PMN interaction with pulmonary MVEC (PMVEC) has been shown to exacerbate the observed barrier dysfunction in response to stimulation with a mixture of pro-inflammatory cytokines (TNFα, IL1β, and IFNγ) and this is thought to be due, at least in part, to PMN-derived NO (8, 22). Further evidence supporting the critical function for PMN in driving septic pulmonary microvascular permeability was provided by *in vivo* murine studies in which PMN were removed (9). Specifically, septic pulmonary microvascular permeability was found to be completely abrogated in septic mice *in vivo* in the absence of PMN or following antibody-mediated blocking of the CD18-β2 integrin adhesion pathway (9).

Apoptosis is a tightly regulated form of cell death known to be important both in tissue homeostasis and under pathological conditions (i.e., sepsis). One of the hallmarks of apoptosis is activation of a family of cysteine proteases known as caspases (23, 24). In addition to caspase activation, apoptotic cell death is also associated with a loss of cell membrane polarization leading to phosphatidyl serine appearing on the outer leaflet of the cell membrane, and fragmentation of the DNA leading to condensed nuclei (23, 24).

Our previous *in vivo* studies in mice revealed both a temporal association and a strong correlation between septic PMVEC apoptosis and PMVEC barrier dysfunction, and importantly, this PMVEC apoptosis appeared to be dependent on PMN–PMVEC interaction (9, 25). However, further assessment of the role of apoptosis in septic murine PMVEC barrier dysfunction *in vitro* demonstrated that early septic barrier dysfunction in PMVEC cultured alone did not appear to be associated with PMVEC apoptosis (26). This finding was supported by multiple other studies that suggested that the connection between MVEC apoptosis and septic barrier dysfunction is unclear and appears to depend on MVEC type, as well as the specific stimulation and time course (26–31). Collectively, these studies highlight the importance of further studies examining the mechanisms responsible for septic MVEC barrier dysfunction, including more clinically relevant direct examination of human MVEC dysfunction and the specific contribution of PMN.

## Materials and Methods

### Reagents

Calcein-AM or calcein-red: Thermo Fisher Scientific (Burlington, ON, Canada); Caspase 1 inhibitor (Ac-YVAD-CMK): Sigma (Oakville, ON, Canada); Caspase 3 inhibitor (Z-DEVD-FMK): APExBIO (Boston, MA, USA); Caspase 8 inhibitor (Z-IETD-FMK): MBL International Corporation (Woburn, MA, USA); Caspase 9 inhibitor (Z-LEHD-FMK): MBL International Corporation (Woburn, MA, USA); CD31 Dynabeads: Life Technologies AS (Oslo, MN, USA); Endothelial Cell Growth Medium-2 (EGM-2): Lonza (Walkersville, MD, USA); Evans blue (EB)-labeled bovine serum albumin (BSA): Sigma (Oakville, ON, Canada); fluoroisothiocyanate (FITC) or Alexa Fluor 647-labeled Annexin V: BioLegend (San Diego, CA, USA); FITC-labeled dextran: Sigma (Oakville, ON, Canada); Human TNFα, IL1β, IFNγ: PeproTech (Rocky Hill, NJ, USA); Lymphocyte Separation Medium (LSM): MP Biomedical (Canada); Pan-caspase inhibitor (Q-VD-OPh hydrate): APExBIO (Boston, MA, USA); Sulforhodamine (SR) FLICA Poly Caspase Assay Kit: Immunohistochemistry Technologies (Bloomington, MN, USA); terminal deoxynucleotidyl transferase dUTP nick end labeling (TUNEL) *In Situ* Cell Death Detection kit: Roche (Laval, QC, Canada); Type II collagenase: Worthington Biochemical Corporation (Lakewood, NJ, USA).

### Isolation and Culture of Human PMVEC

PMVEC were isolated from human lung as previously reported (22). Briefly, human peripheral lung tissue isolated from a grossly normal-appearing region obtained during resectional surgery for localized lung cancer was rinsed in PBS, finely minced, and digested in 0.3% type II collagenase at 37°C with occasional agitation. The digested suspension was filtered, centrifuged at 200 *g*, and washed in PBS. The cell pellet was then resuspended in binding buffer (2 μM Na-citrate, 1.2 μM NaH_2_PO_4·_H_2_O, 5.6 μM Na_2_HPO_4_, 138.6 μM NaCl, and 0.1% BSA) and incubated at 4°C with magnetic Dynabeads coated with anti-human CD31 antibody. Bound cells were magnetically isolated and washed with binding buffer. Isolated cells were resuspended in 10% EGM-2 and placed at 37°C in 5% CO_2_ until 50% confluent. PMVEC were monitored daily and quickly growing fibroblasts were removed mechanically under direct microscopy. Once PMVEC reached 50% confluence, they were harvested and re-purified using anti-CD31-coated magnetic microbeads as above. PMVEC were used for experiments at passages 4–10.

### Isolation and Labeling of Human Blood Polymorphonuclear Leukocytes (PMN)

PMN were isolated from healthy human blood donors using LSM as described previously (22, 32). In some experiments, PMN were labeled with calcein-AM or calcein-red according to the manufacturer’s instructions.

### Experimental Conditions

PMVEC medium was changed to fresh 10% EGM-2 at 16 h before PMVEC stimulation. For PMVEC cultured alone, cytomix (equimolar human TNFα, IL1β, and IFNγ in PBS) was added to PMVEC at a final concentration of 30 ng/mL (vs. an equal volume of PBS control). For PMN–PMVEC coculture experiments, PMN and PMVEC were individually stimulated with cytomix at 0.3 and 3 ng/mL vs. PBS control (Figure S1 in Supplementary Material). After 3 h, PMN were added to the corresponding PMVEC monolayer (PMN:PMVEC ratio = 10:1), and cocultured together with or without cytomix stimulation for another 2 h (Figure S1 in Supplementary Material). Isolated PMVEC were treated with caspase inhibitors (Q-VD, Ac-YVAD-CMK, Z-DEVD-FMK, Z-IETD-FMK, and Z-LEHD-FMK) for the entire duration (5 h) of the study, but for PMVEC–PMN coculture studies, caspase inhibitors were applied to PMVEC for the 3 h prior to coculture and then for the 2 h of coculture.

### Assessment of PMVEC Barrier Function

PMVEC were seeded at a concentration of 10^4^ cells/well on gelatin-coated 24-well cell-culture inserts (3.0 μm pore, VWR Scientific) in 10% EGM-2 as we have done previously (33). Once confluent, PMVEC monolayer permeability was assessed by (1) transendothelial electrical resistance (TEER; EVOM2 Endothelial Voltohmmeter; World Precision Instruments, Sarasota, FL, USA); (2) FITC-labeled dextran flux (FITC-dextran, 4 kDa); and (3) EB-labeled BSA flux (EB-BSA, 67 kDa). Trans-PMVEC macromolecular flux from the upper chamber into the lower chamber of the cell-culture inserts was measured over exactly 1 h (Figure S1 in Supplementary Material). Briefly, 50 μL of EB-BSA (containing 33.5 μg of EB) and FITC-dextran (125 μg) was added gently to the upper chamber (final volume 250 μL) of the cell-culture insert at 1 h before each indicated time point of assessment. At the same time, 150 μL of the same concentration of unlabeled BSA was added to the lower chamber. After exactly 1 h, inserts were removed and the media from the lower chamber collected. EB-BSA flux was determined by measuring absorbance (620 nm) and FITC-dextran flux was determined by measuring fluorescence (excitation 488 nm and emission 525 nm; Victor3 microplate reader, PerkinElmer, Woodbridge, ON, Canada). All leak measures (TEER, EB-BSA, and dextran) were normalized to a blank well containing only an insert with no PMVEC present.

### Assessment of PMVEC Apoptosis

PMVEC apoptosis was detected by measuring caspase activation, loss of cell membrane polarization, and DNA fragmentation as we previously described (26). To detect caspase activation, PMVEC were stained with the FLICA Poly Caspase Assay Kit as per the manufacturer’s instructions. For PMN–PMVEC coculture experiments, PMN were pre-labeled with calcein-AM to identify them during fluorescence microscopy. PMVEC were then fixed with methanol and Hoechst stain was used to identify nuclei. Cells were then imaged using fluorescent microscopy (FLICA excitation/emission: 550/590–600 nm; Hoechst excitation/emission: 361/486 nm). The number of FLICA and Hoechst positive cells per field of view was assessed through manual counting (two blinded reviewers) and automated counting using ImageJ (National Institutes of Health).

Loss of cell membrane polarization (as indicated by presence of cell surface phosphatidylserine) was assessed by staining PMVEC with FITC-conjugated Annexin V and propidium iodide (PI). Following stimulation with PBS or cytomix, the medium containing detached cells was collected, and the remaining attached cells lifted by Accutase. Both detached and attached PMVEC populations were pooled and stained with Annexin V and PI in binding buffer (0.1 M HEPES pH 7.4; 140 mM NaCl; 25 mM CaCl_2_). PMVEC were then analyzed by flowcytometry (easyCyte Guava 12HT). Annexin V+/PI− cells were considered early-phase apoptotic cells, whereas Annexin V+/PI+ cells were considered dead cells and Annexin V−/PI− cells were considered live cells.

Late-stage apoptotic DNA fragmentation in PMVEC was examined by analysis of terminal deoxynucleotidyl TUNEL using the *In Situ* Cell Death Detection kit. For these studies, PMVEC were fixed in methanol following stimulation and then permeabilized with a 0.1% Triton X-100 solution. Following permeabilization, TUNEL staining was used to identify PMVEC with DNA fragmentation and Hoechst stain was used to label all PMVEC nuclei. Cells were imaged and the number of TUNEL and Hoechst positive cells per field of view was determined as above. In addition, for all FLICA and TUNEL experiments, all detached cells were collected and stained as above, cytospun onto a slide, and images captured.

### Statistics

Data are reported as mean ± SEM and were analyzed using GraphPad Prism 5. Differences between groups were assessed by *t*-tests (one measured variable) or by a two-way ANOVA with Bonferroni *post hoc* testing (two independent variables). Significance threshold was set at α = 0.05 and experiments were replicated at least three times using three different primary human PMVEC. The correlation of septic PMVEC permeability (EB-BSA flux) with caspase activation (FLICA+ EC) was assessed by Pearson’s method.

## Results

### Effects of Septic (Cytomix) Treatment on Human PMVEC Barrier Function and Apoptosis

**Figure 1:**
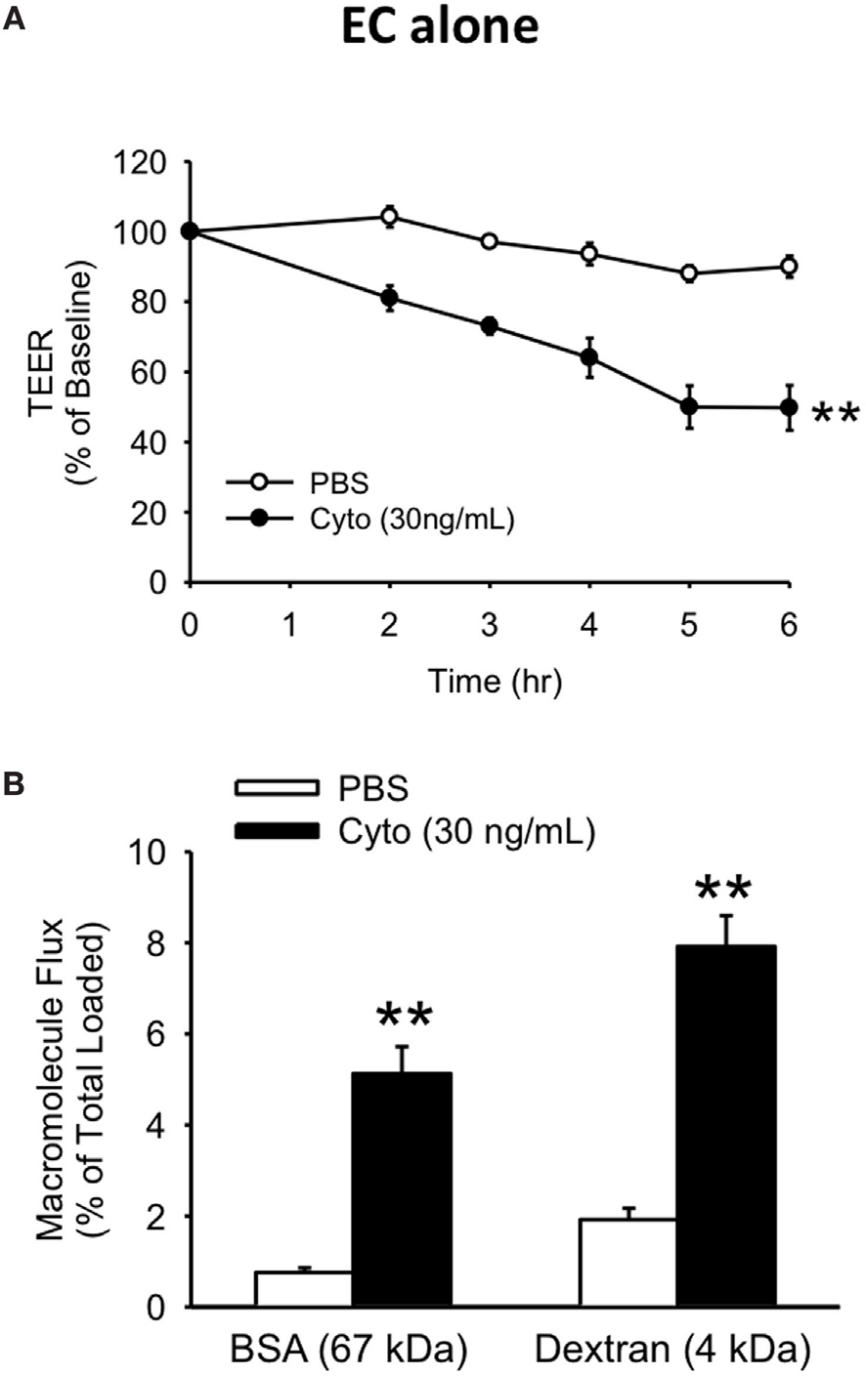
Cytomix induces significant loss of human PMVEC barrier function. Cytomix-stimulated PMVEC cultured alone had significantly increased permeability that peaked by 5 h post-stimulation and persisted at 6 h post-stimulation vs. PBS by three assays: lower transendothelial electrical resistance (TEER) **(A)** and higher macromolecular flux including Evans blue-bovine serum albumin (BSA) and fluoroisothiocyanate-dextran **(B)**. **p < 0.01 compared with respective PBS (repeated measures two-way ANOVA or *t*-test). *n* = 6. Note: for the sake of simplicity, the term EC refers specifically to PMVEC within all figures.

To examine the relationship between human PMVEC barrier dysfunction and apoptosis under septic conditions, we initially characterized the time course of septic (cytomix 30 ng/mL) stimulation-induced barrier dysfunction of human PMVEC monolayers cultured alone over 6 h. In our previous dose-response studies, this was the maximally effective cytomix dose that did not induce massive loss of PMVEC viability (22). PMVEC permeability was assessed by measuring trans-PMVEC ion movement using TEER. TEER was significantly reduced following cytomix stimulation of PMVEC compared to PBS-treated PMVEC, with the greatest decrease in TEER occurring by 5–6 h post-stimulation, indicating peak permeability (Figure 1A).

Based on the time course of septic changes in TEER, we subsequently assessed specifically at 5 h post-cytomix treatment changes in PMVEC permeability to macromolecules, of greater relevance to human sepsis and ARDS, including EB-BSA (67 kDa) and FITC-dextran (4 kDa). Septic PMVEC barrier dysfunction was confirmed by significant increases in both BSA and dextran flux at 5 h post-cytomix stimulation vs. PBS treatment (Figure 1B). Basal trans-PMVEC flux of the smaller dextran molecule was significantly greater than the flux of the larger BSA (1.92 ± 0.10% of total dextran loaded vs. 0.76 ± 0.04% of total BSA loaded, respectively, *p* < 0.05). Moreover, this higher dextran flux vs. BSA flux under basal conditions resulted in the relative cytomix-induced increase being greater for BSA than for dextran (777 ± 116% of PBS-treated vs. 454 ± 47% of PBS-treated for BSA and dextran, respectively, *p* < 0.05).

**Figure 2:**
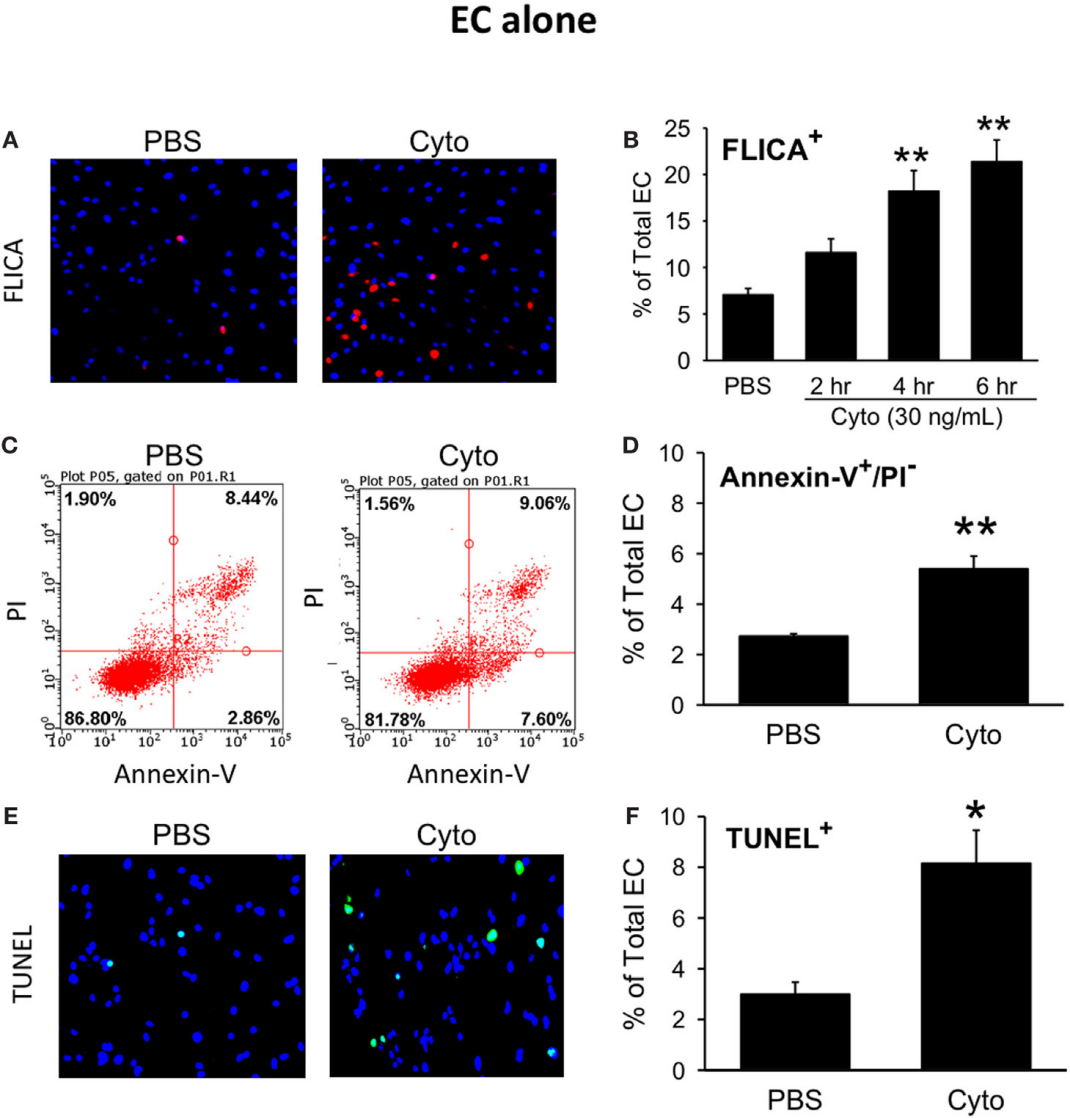
Cytomix induces significant human PMVEC apoptosis. Cytomix stimulation of PMVEC cultured alone leads to an increased number of cells stained positive for **(A)** active caspases [fluorescent inhibitor of caspases (FLICA); red]; **(C)** loss of cell membrane polarization (Annexin V); and **(E)** DNA fragmentation [terminal deoxynucleotidyl transferase dUTP nick end labeling (TUNEL); green]. Quantification revealed significant increases in FLICA+ cells **(B)** by 4 h post-cytomix that persisted at 6 h post-cytomix. The number of Annexin-V+/propidium iodide (PI)− cells **(D)** and TUNEL+ cells **(F)** were also significantly increased by 5 h post-cytomix. *p < 0.05 and **p < 0.01 compared with respective PBS-treated group (two-way ANOVA or *t*-test). *n* = 4–7.

We next assessed the effects of septic treatment of human PMVEC on markers of PMVEC apoptosis. Caspase activation, one of the molecular features of apoptosis, was assessed using the fluorescent marker FLICA. We observed a significant increase in the number of FLICA+ PMVEC by 4 h post-cytomix vs. PBS, that remained similarly increased at 6 h (Figures 2A,B). Based on the time course of changes in caspase activation, we also assessed two other markers of PMVEC apoptosis specifically at 5 h post-cytomix treatment, including changes in PMVEC membrane polarization (Annexin V staining) and DNA fragmentation (TUNEL). We observed significant increases in both Annexin V+ (Figures 2C,D) and TUNEL+ (Figures 2E,F) PMVEC following cytomix stimulation vs. PBS.

### Effect of Caspase Inhibition on Cytomix-Induced Permeability and Apoptosis in Human PMVEC Cultured Alone

**Figure 3:**
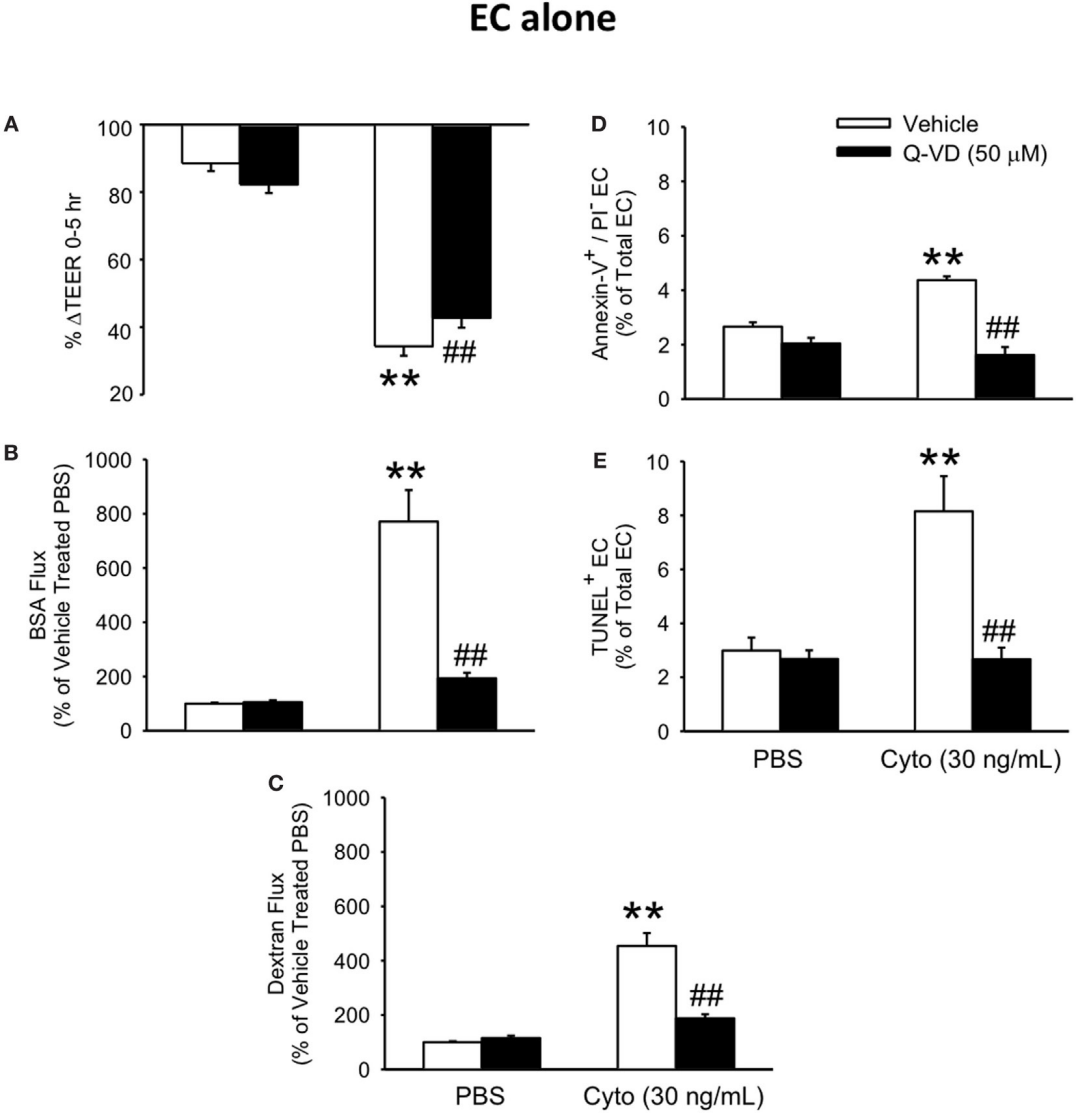
Inhibition of caspase activity reduces septic human PMVEC hyper-permeability and the associated apoptosis. Inhibition of caspase activity (Q-VD, 50 μM) following cytomix stimulation of human PMVEC cultured alone modestly attenuated the septic decrease in transendothelial electrical resistance (TEER) **(A)** but more markedly blunted the septic increases in macromolecular flux, including Evans blue-albumin **(B)** and fluoroisothiocyanate-dextran **(C)** vs. vehicle treatment (dimethyl sulfoxide). Furthermore, treatment of human PMVEC cultured alone with Q-VD also completely prevented the septic increase in the number of Annexin-V+/propidium iodide (PI)− **(D)** and TUNEL+ **(E)** cells. ** or ^##^*p* < 0.01 compared with cytomix or vehicle group, respectively (two-way ANOVA). *n* = 4–6.

To determine the contribution of PMVEC apoptosis to septic PMVEC barrier dysfunction, human PMVEC were cultured in the presence or absence of Q-VD, a pan-caspase inhibitor previously found to inhibit PMVEC apoptosis (26). Treatment of cytomix-stimulated PMVEC with Q-VD led to a significant restoration of PMVEC barrier function. Specifically, Q-VD treatment slightly attenuated the septic fall in PMVEC TEER (Figure 3A), but dramatically blunted the septic increases in trans-PMVEC macromolecule flux of both EB-BSA and FITC-dextran, compared to vehicle-treated septic PMVEC (Figures 3B,C). Furthermore, treatment of PMVEC with Q-VD completely abrogated the cytomix-induced increases in Annexin V+ and TUNEL+ PMVEC vs. vehicle treatment (Figures 3D,E).

### Effects of Septic (Cytomix) Treatment on Human PMVEC Barrier Function and Apoptosis in the Presence of PMN

**Figure 4:**
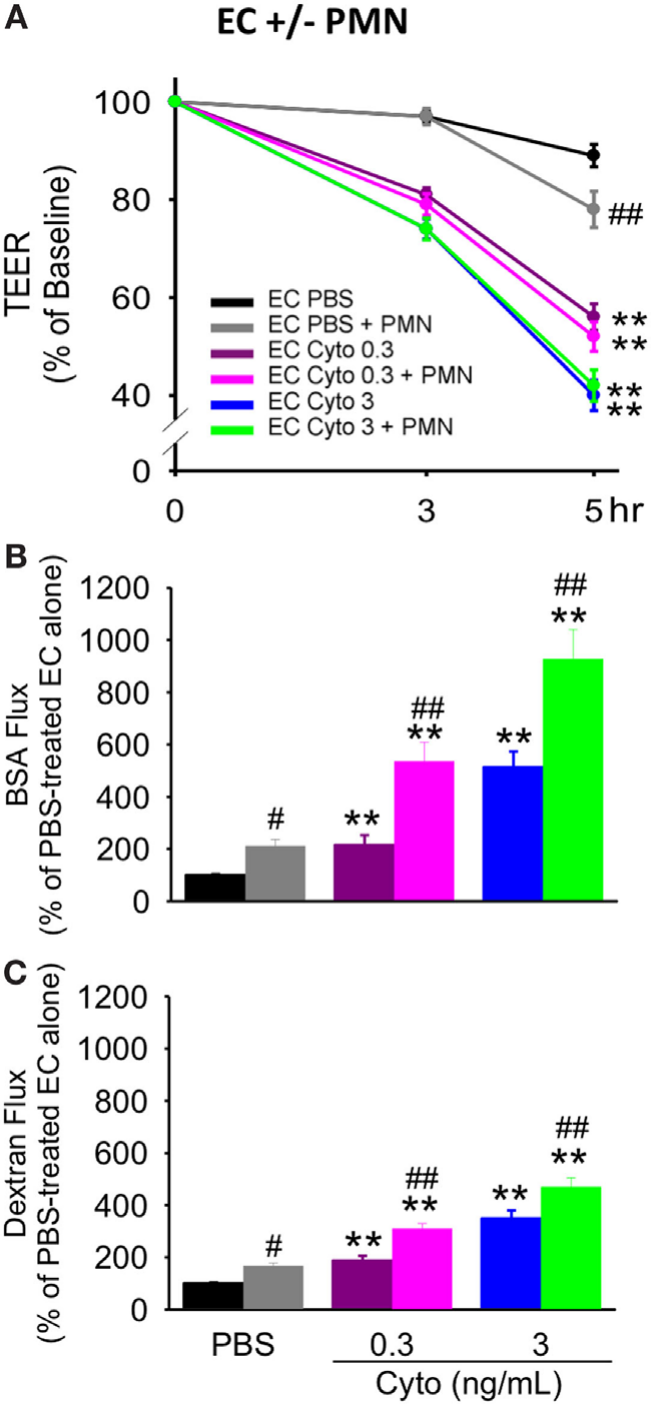
PMN coculture with PMVEC significantly augments septic trans-PMVEC macromolecular flux. Compared to human PMVEC alone, coculture of PMVEC with PMN led to significantly increased leak under basal (PBS) conditions, as assessed by three measures: transendothelial electrical resistance (TEER) **(A)**, Evans blue (EB)-bovine serum albumin (BSA) **(B)**, and fluoroisothiocyanate (FITC)-dextran **(C)**. Cytomix treatment (0.3 and 3 ng/mL) of PMVEC cultured alone or in coculture with PMN increased permeability, including decreased TEER and increased EB-BSA and FITC-dextran flux. However, under septic conditions, PMN–PMVEC coculture only significantly augmented macromolecular flux **(B,C)** compared to PMVEC alone. **p < 0.01 compared with respective PBS-treated group; ^#^*p* < 0.05 and ^##^*p* < 0.01 for PMN + PMVEC compared with PMVEC alone (two-way ANOVA). *n* = 6.

We have previously shown that PMN contribute to septic PMVEC barrier dysfunction and apoptosis in murine sepsis *in vivo* (9). To assess a possible contribution of human PMN to septic human PMVEC barrier dysfunction and apoptosis, PMVEC were cocultured with PMN under basal (PBS) and septic (cytomix) conditions. Based on our previous studies, 30 ng/mL cytomix is an excessive stimulus for PMN–PMVEC cocultures, leading to massive loss of cell viability (22). As such, two lower cytomix doses were used for septic PMN–PMVEC coculture studies, low (0.3 ng/mL) and medium-dose (3 ng/mL) cytomix treatment. The presence of PMN slightly decreased PMVEC barrier function under basal conditions compared to PMVEC cultured alone, as indicated by significantly lower TEER (Figure 4A) and significantly higher EB-BSA and FITC-dextran flux (Figures 4B,C).

Although basal PMVEC TEER was lower in PMN–PMVEC coculture than in PMVEC alone, the presence of PMN had no effect on the septic decrease in PMVEC TEER vs. PMVEC cultured alone, at both low and medium cytomix doses (Figure 4A). By contrast, coculture of PMVEC with PMN significantly enhanced septic PMVEC barrier dysfunction (increases in both EB-BSA and FITC-dextran flux) vs. PMVEC cultured alone, at both low and medium-dose cytomix (Figures 4B,C).

**Figure 5:**
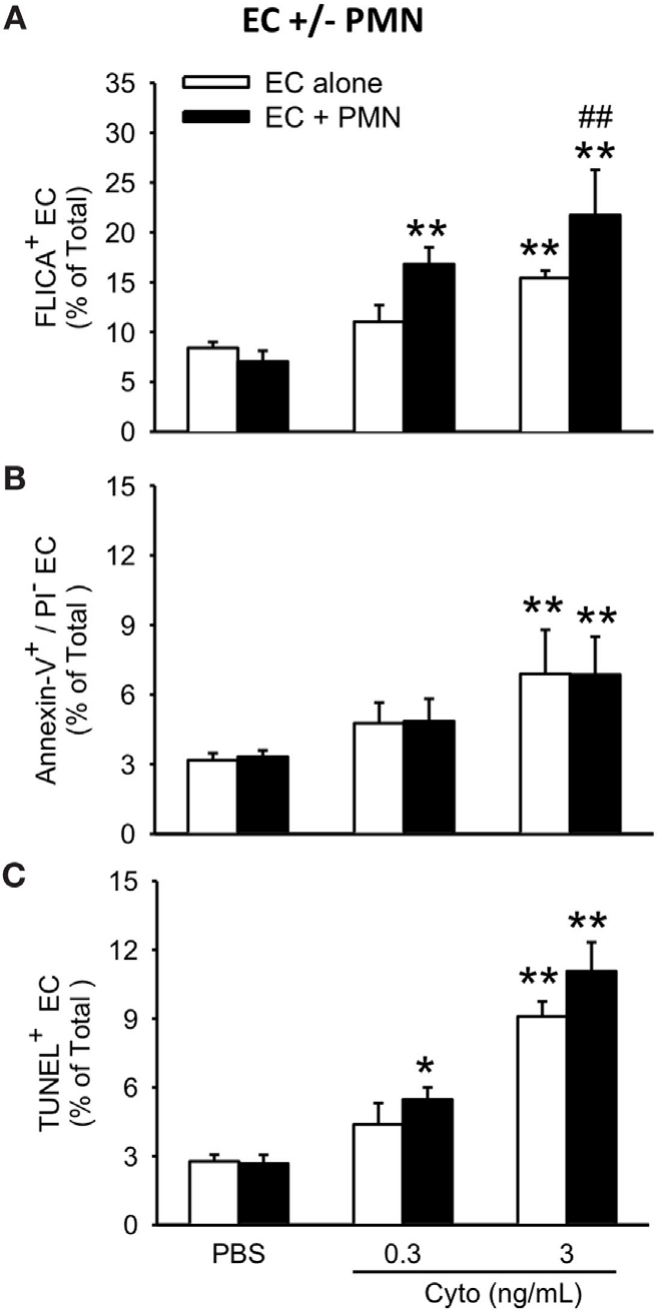
PMN coculture with PMVEC increases septic caspase activation but does not augment septic PMVEC apoptosis. In isolated PMVEC, only moderate-dose cytomix (3 ng/mL), but not low-dose (0.3 ng/mL), increased the number of apoptotic PMVEC as indicated by three complementary measures: **(A)** FLICA+ cells, **(B)** Annexin-V+/propidium iodide (PI)− cells, and **(C)** TUNEL+ cells. Cytomix stimulation of PMN–PMVEC cocultures increased PMVEC caspase activation (FLICA+) at both low- and moderate-doses but had negligible effects on other markers of apoptosis (Annexin-V+/PI− and TUNEL+). *p < 0.05 and **p < 0.01 compared with respective PBS-treated group; ^##^*p* < 0.01 for PMN + PMVEC compared with PMVEC alone (two-way ANOVA). *n* = 3–4.

To assess the contribution of PMVEC apoptosis to PMN-enhanced septic PMVEC barrier dysfunction, markers of apoptosis were examined in PMVEC cocultured with PMN under basal and septic conditions. Under basal conditions, the presence of PMN had no effect on PMVEC apoptosis vs. PMVEC cultured alone, as evidenced by all three measures of apoptosis (FLICA, Annexin V, and TUNEL; Figure 5). In PMVEC cultured alone, medium-dose cytomix stimulation (3 ng/mL) induced septic PMVEC apoptosis, but not low-dose cytomix stimulation (0.3 ng/mL; Figure 5). Compared to cytomix-treated PMVEC alone, the presence of PMN-enhanced septic PMVEC FLICA staining (this achieved significance at a medium cytomix dose, 3 ng/mL; Figure 5A), but PMN presence had no effect on the septic increases in other markers of PMVEC apoptosis, including both Annexin V and TUNEL staining (Figures 5B,C).

**Figure 6:**
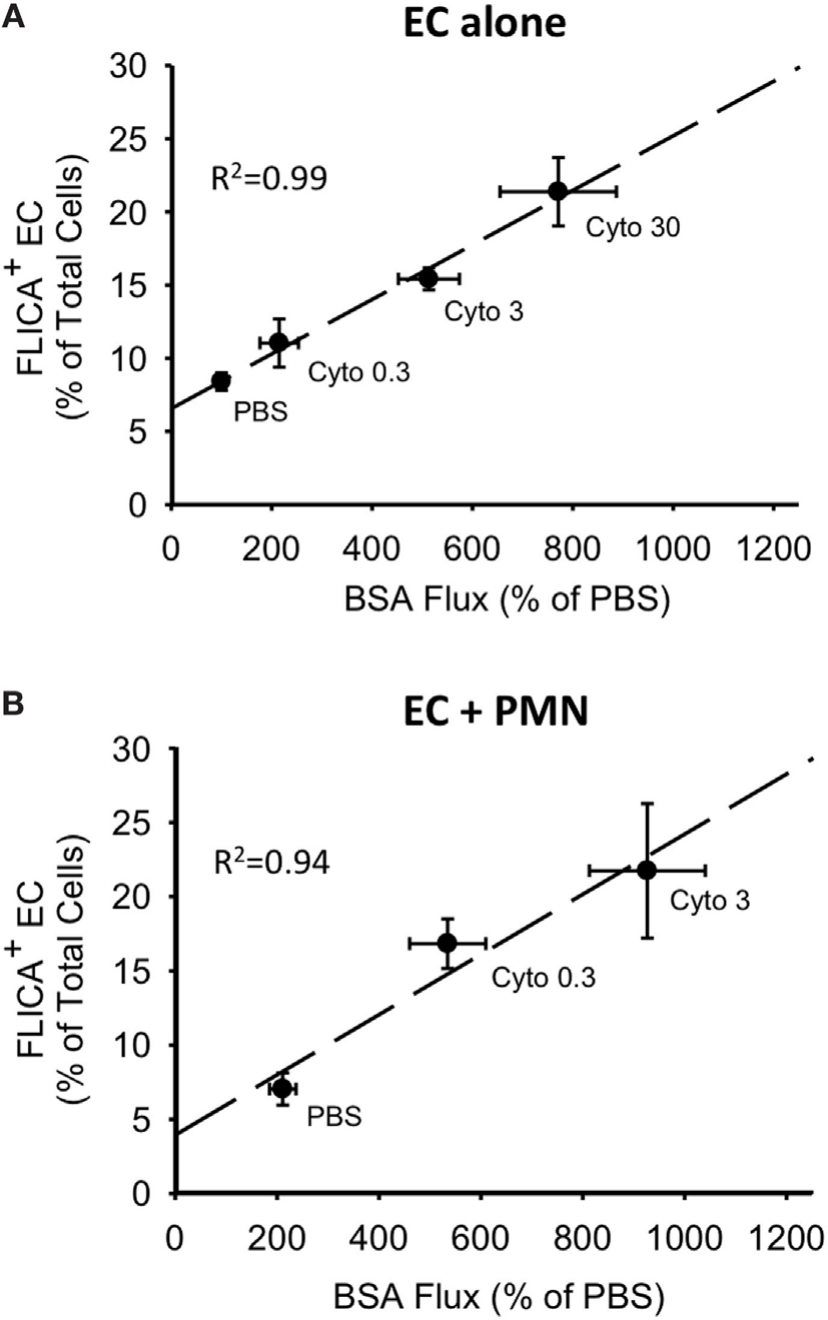
Correlations between cytomix-induced increases in PMVEC caspase activity and trans-PMVEC macromolecular flux in isolated PMVEC and in PMN–PMVEC cocultures. Cytomix dose-dependently increased PMVEC macromolecular permeability [Evans blue-bovine serum albumin (BSA); *x*-axis], which was highly correlated with caspase activation (FLICA+ cells; *y*-axis) in PMVEC cultured alone **(A)** and in coculture with PMN **(B)**. Note: given the greater sensitivity of PMN–PMVEC cocultures **(B)** to cytomix stimulation, only low (0.3 ng/mL) and medium (3 ng/mL) doses of cytomix were used, compared to high dose (30 ng/mL) cytomix in PMVEC alone **(A)**. *n* = 4–6 for each dose.

For both PMVEC cultured alone and PMVEC cocultured with PMN, septic PMVEC barrier dysfunction, as reflected by increased EB-BSA flux, was strongly correlated with PMVEC caspase activation, as indicated by an increased number of FLICA+ PMVEC (Figures 6A,B). Although the percentage increase in trans-PMVEC flux of dextran was smaller, because of higher basal flux of the smaller dextran molecule, compressing the septic signal, there were also significant correlations between PMVEC FLICA staining and trans-PMVEC dextran flux in both isolated PMVEC and in PMN–PMVEC coculture (data not shown).

### Effect of Caspase Inhibition on Cytomix-Induced Permeability and Apoptosis in Human PMN–PMVEC-Coculture

**Figure 7:**
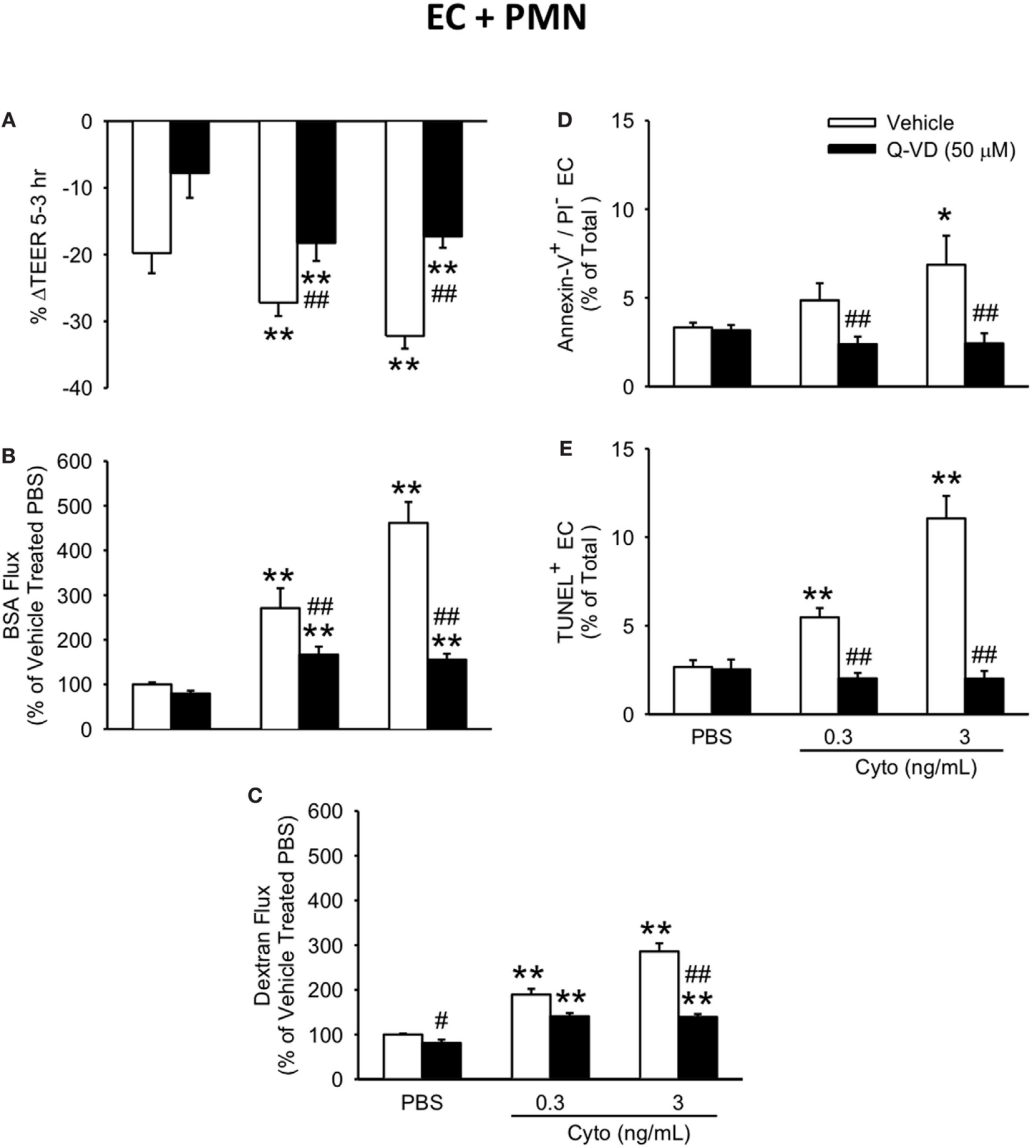
Inhibition of caspase activity in human PMVEC cocultured with PMN reduces septic hyper-permeability and apoptosis. Treatment of PMN–PMVEC cocultures with Q-VD (50 μM) significantly attenuated the septic decreases in transendothelial electrical resistance (TEER) **(A)** as well as the septic increases in macromolecular flux, including Evans blue-bovine serum albumin (BSA) **(B)** and fluoroisothiocyanate-dextran **(C)** vs. vehicle treatment (dimethyl sulfoxide). Furthermore, Q-VD treatment of PMVEC cultured with PMN also significantly blunted the septic increase in the number of Annexin-V+/propidium iodide (PI)− **(D)** and TUNEL+ **(E)** cells. *p < 0.05 and **p < 0.01 compared with respective PBS-treated group; ^##^*p* < 0.01 compared with respective vehicle-treated group (two-way ANOVA). *n* = 3–6.

To determine the contribution of caspase activation to PMN-enhanced septic PMVEC barrier dysfunction, human PMN–PMVEC cocultures were treated with Q-VD vs. dimethyl sulfoxide vehicle. Q-VD treatment of PMVEC cocultured with PMN led to a partial, but significant restoration of PMVEC barrier function (Figure 7). Specifically, treatment with Q-VD significantly attenuated the septic decrease in PMVEC TEER compared to vehicle-treated PMVEC (Figure 7A). Moreover, Q-VD treatment also significantly blunted the increased trans-PMVEC macromolecular flux across PMVEC cocultured with PMN under septic conditions, including both EB-BSA (Figure 7B) and FITC-dextran (Figure 7C). Furthermore, the cytomix-induced increase in trans-PMVEC macromolecular flux across PMVEC cultured alone or in coculture with PMN was associated with the formation of PMVEC intercellular gaps and treatment with Q-VD significantly blunted this gap formation (Figure S2 and Materials and Methods of Data Sheet 1 in Supplementary Material).

**Figure 8:**
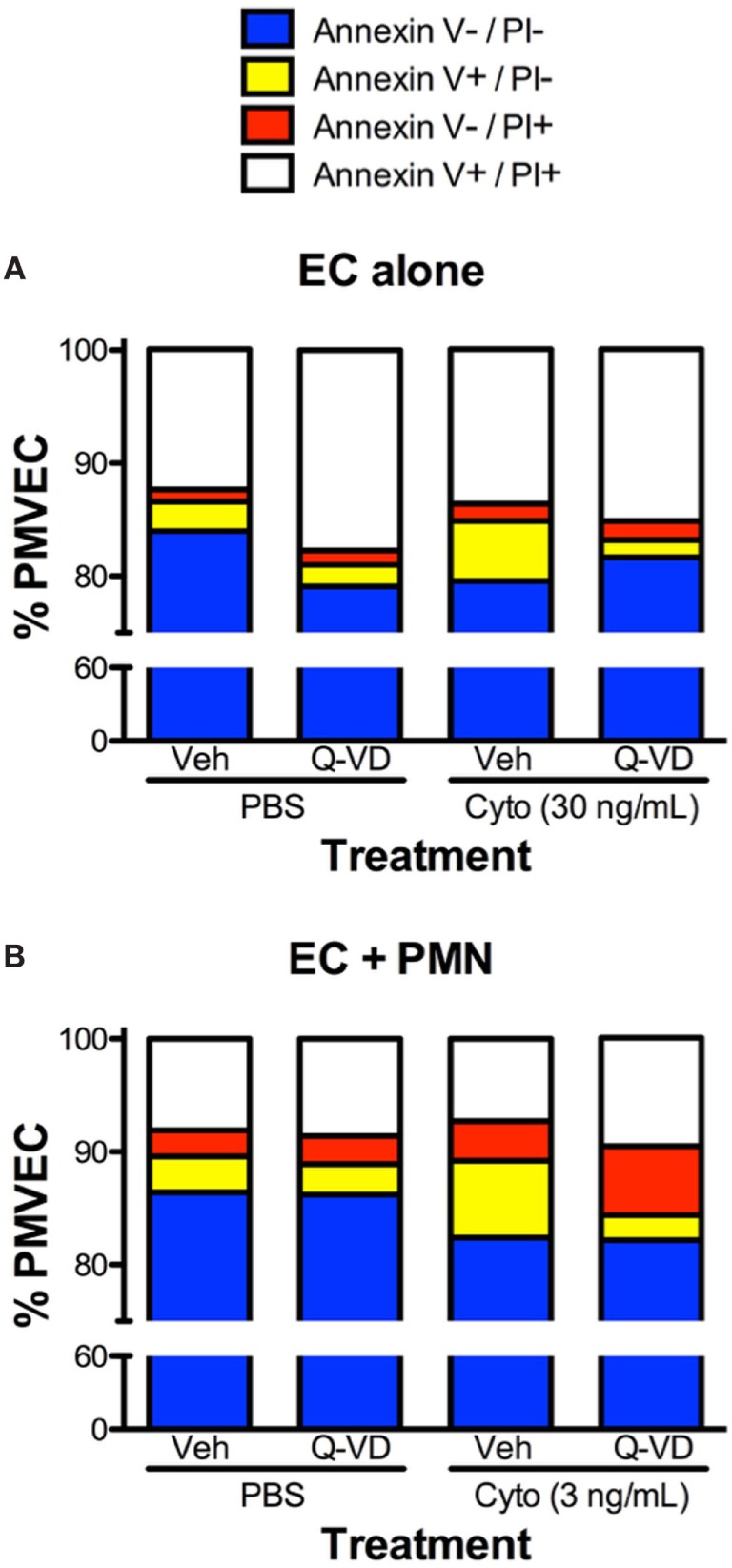
Effects of cytomix treatment of human PMVEC on different types of cell death. **(A)** Cytomix (30 ng/mL) treatment increased Annexin V+/propidium iodide (PI)− PMVEC (yellow bar; suggestive of early apoptosis), but had no effect on PI staining, including specifically both Annexin V−/PI+ cells (red bar; indicative of pyroptosis) and Annexin V+/PI+ cells (white bar; later stage apoptosis and necrosis/necroptosis). The cytomix-induced increase in Annexin V staining was completely abrogated by Q-VD treatment. **(B)** Similarly, in PMN–PMVEC cocultures, cytomix treatment (3 ng/mL) increased Annexin V+/PI− PMVEC (yellow bar) and was not associated with any significant change in Annexin V−/PI+ (red bar) and Annexin V+/PI+ (white bar) PMVEC vs. PBS. The cytomix-induced increase in Annexin V+/PI− PMVEC was completely inhibited by Q-VD treatment. Complete data and variance are provided in Table 1.

In addition to blunting cytomix-induced macromolecular flux, Q-VD treatment of PMVEC cocultured with PMN also significantly reduced the sepsis-induced increases in Annexin V+ and TUNEL+ PMVEC vs. vehicle-treated (Figures 7D,E). To assess the potential contribution of other cell death sub-types, such as necroptosis and necrosis, further analysis of Annexin V and PI staining of PMVEC cultured alone or in coculture with PMN was performed. Cytomix stimulation or treatment with Q-VD had no effect on PI staining, including the percentage of Annexin V−/PI+ or Annexin V+/PI+ PMVEC (Figure 8; Table 1). Importantly, while the percentage of Annexin V−/PI+ PMVEC appeared to increase following Q-VD treatment of PMVEC stimulated with cytomix in coculture with PMN, these differences did not reach significance (Figure 8; Table 1).

**Table 1:** Effects of cytomix and Q-VD treatment on different types of cell death in isolated human PMVEC and in PMN–PMVEC cocultures.

Group/treatment	Annexin V−/propidium iodide (PI)− (%)	Annexin V+/PI− (%)	Annexin V−/PI+ (%)	Annexin V+/PI+ (%)
**EC alone**				
PBS	84.0 ± 2.3	2.6 ± 0.1	1.1 ± 0.3	12.4 ± 2.2
PBS + Q-VD	79.1 ± 1.6	1.9 ± 0.2	1.3 ± 0.2	17.7 ± 1.8
Cytomix (30 ng/mL)	79.6 ± 1.6	5.3 ± 0.5**	1.5 ± 0.3	13.7 ± 1.8
Cytomix + Q-VD	81.7 ± 1.7	1.5 ± 0.2^##^	1.7 ± 0.2	15.2 ± 1.5
**EC + PMN**				
PBS	86.4 ± 0.4	3.2 ± 0.2	2.3 ± 0.2	8.1 ± 0.5
PBS + Q-VD	86.2 ± 1.0	2.7 ± 0.1	2.5 ± 0.6	8.6 ± 1.0
Cytomix (3 ng/mL)	82.4 ± 0.5	6.8 ± 1.4*	3.5 ± 0.2	7.3 ± 1.8
Cytomix + Q-VD	82.2 ± 1.9	2.2 ± 0.6^##^	6.1 ± 1.8	9.6 ± 1.1

*One-way ANOVA with Tukey HSD post hoc test for each column*.

*p < 0.05 and **p < 0.01 for cytomix vs. respective PBS.

*^##^p < 0.01 for Q-VD-treated vs. cytomix*.

*n = 3–6*.

**Figure 9:**
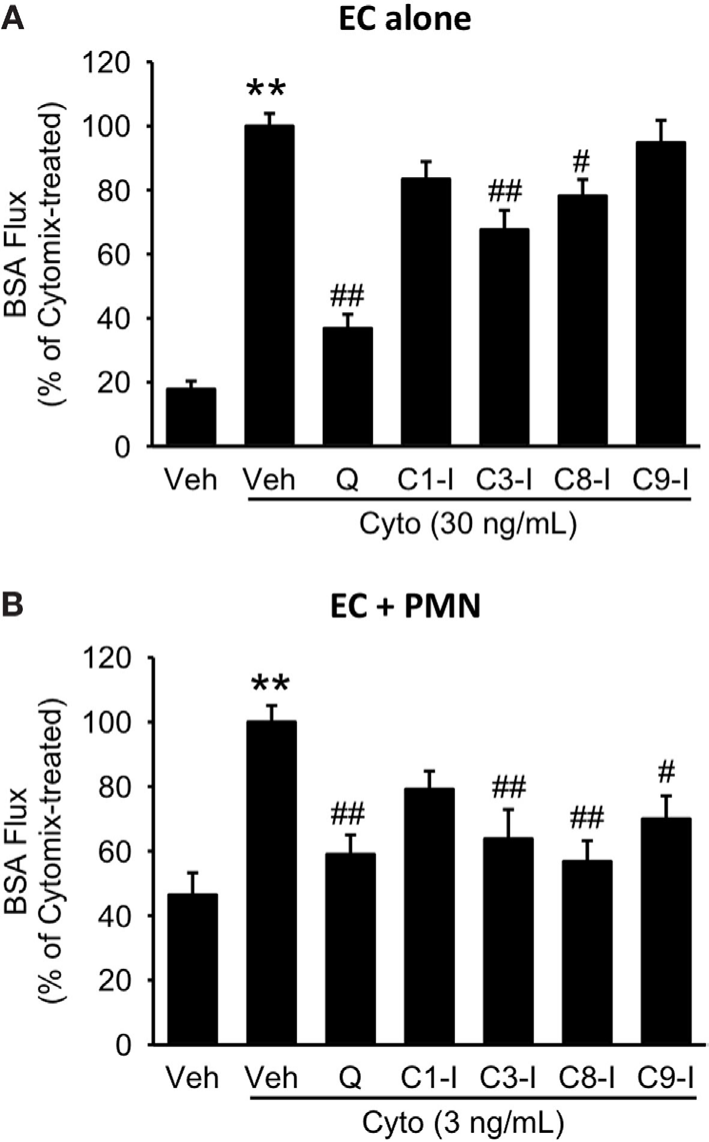
Effect of pan-caspase vs. selective caspase inhibition on septic hyper-permeability in human PMVEC alone or cocultured with PMN. **(A)** In isolated PMVEC, pan-caspase inhibition with Q-VD treatment markedly inhibited cytomix-induced trans-PMVEC bovine serum albumin (BSA) flux. Inhibition of caspase3 (C3-I) and caspase8 (C8-I) had a similar but smaller inhibitory effect on septic PMVEC hyper-permeability, but inhibition of caspase1 (C1-I) or caspase9 (C9-I) had no effect. **(B)** In PMN–PMVEC cocultures, pan-caspase inhibition with Q-VD almost completely inhibited septic PMVEC hyper-permeability, with similar effects of individual caspase3, caspase8, and caspase9 inhibition, and no effect on septic PMVEC hyper-permeability of inhibition of caspase1. **p < 0.01 for cytomix vs. non-cytomix treatment; ^#^*p* < 0.05 and ^##^*p* < 0.01 for caspase inhibitor treatments vs. cytomix alone (one-way ANOVA). *n* = 7–8.

To further define the role of caspase activity in septic PMVEC barrier dysfunction, human PMVEC cultured alone or in coculture with PMVEC were treated with inhibitors against specific caspases, including caspase 1, 3, 8, and 9. In PMVEC cultured alone, inhibition of caspase 3 and 8 significantly attenuated cytomix-induced trans-PMVEC EB-BSA flux while inhibition of caspase 1 and 9 had no significant impact (Figure 9A). Similarly, treatment with caspase 3 or 8 inhibitors of PMN–PMVEC cocultures significantly reduced cytomix-stimulated EB-BSA flux while inhibition of caspase 1 had no effect (Figure 9B). However, unlike PMVEC cultured alone (Figure 9A), treatment of PMVEC cocultured with PMN with a caspase 9 inhibitor significantly reduced cytomix-stimulated EB-BSA flux (Figure 9B).

## Discussion

In the present report, isolated human PMVEC were cultured either alone or in the presence of human PMN, under septic conditions induced by exposure to multiple sepsis-relevant pro-inflammatory cytokines, as an *in vitro* model of human sepsis. Septic stimulation of isolated human PMVEC resulted in significant barrier dysfunction associated with increased PMVEC presence of three markers of apoptosis, including FLICA (caspase activation), Annexin V (cell surface phosphatidyl serine), and TUNEL (DNA fragmentation) staining. Pan-caspase inhibition with the most potent chemical inhibitor available, Q-VD, in isolated PMVEC expectedly prevented septic PMVEC apoptosis (as evidenced by reduced Annexin V and TUNEL staining), and markedly blunted the septic increase in trans-PMVEC macromolecule flux (both BSA and dextran), but only slightly attenuated the septic decrease in TEER. When human PMN were cocultured with human PMVEC, septic PMVEC barrier dysfunction was exacerbated, and this was associated with greater septic PMVEC caspase activation (FLICA staining). By contrast, PMN-dependent septic PMVEC barrier dysfunction was not associated with any greater degree of septic PMVEC apoptosis (as assessed by both Annexin V and TUNEL staining). Pan-caspase inhibition in PMN–PMVEC cocultures using Q-VD markedly attenuated PMN-dependent septic increases in trans-PMVEC macromolecule flux (both BSA and dextran) but had no effect on septic decreases in TEER. Moreover, inhibition of caspase 3, 8, or 9 in PMN–PMVEC cocultures significantly reduced septic increases in trans-PMVEC EB-BSA flux whereas inhibition of caspase 1 had no effect.

The new definition of human sepsis incorporates the critical idea of septic organ dysfunction (34, 35), which is a primary determinant of the severity of sepsis, and especially of clinical outcomes such as mortality (4). Of all the organs, septic injury of the lung, termed ARDS, is the most common and the most serious in terms of adverse clinical outcomes (36). Organ dysfunction in human sepsis can be related to the actual infection and its dissemination, to the resulting systemic inflammation, and importantly, to organ-specific microvascular dysfunction and injury (11). Septic microvascular dysfunction is clinically relevant, as it is associated with more severe sepsis, greater organ dysfunction, and increased mortality in human sepsis (13, 14, 37, 38). Septic microvascular dysfunction is largely driven by activation and injury specifically of MVEC. For example, increased circulating numbers of EC and higher levels of soluble markers of EC activation/damage [e.g., intercellular adhesion molecule 1, von Willebrand factor, vascular endothelial (VE) growth factor receptor 1] correlate with more severe sepsis and higher mortality (39–45).

Multiple initiating mechanisms for MVEC activation and injury leading to loss of MVEC barrier function in sepsis have been postulated. These include the actions of cytokines and other soluble circulating molecules on MVEC, mechanical interaction of activated leukocytes and platelets with MVEC, and paracrine exposure to injurious molecules released by these circulating cells (8, 16–19, 46). Furthermore, septic MVEC barrier dysfunction and increased paracellular protein-fluid leak can also result from actin cytoskeleton-driven MVEC retraction, signaling-activated cleavage, and/or internalization of cell–cell junctional proteins (e.g., VE-cadherin), which may be associated with altered expression/function of cytosolic adaptor proteins (e.g., β-catenin), or MVEC loss due to cell death and/or disruption of MVEC–matrix interactions (47, 48). The predominant mechanisms of septic PMVEC injury and dysfunction, of greatest direct relevance to human sepsis and ARDS, remain uncertain.

In the current study, we examined septic responses in human PMVEC, because of the clinically important and prognostic role of septic lung dysfunction leading to ARDS in septic humans, the central role of the pulmonary microvasculature in sepsis-associated ARDS, and the direct human relevance of such studies. Specifically, we assessed mechanisms driving septic PMVEC barrier dysfunction under two conditions, the presence or absence of PMN. In isolated PMVEC alone, the loss of PMVEC barrier function is strongly associated with increased PMVEC apoptosis and is caspase-dependent as inhibition of caspases through Q-VD treatment prevented septic PMVEC apoptosis and restored PMVEC barrier function. By contrast, the presence of PMN greatly enhanced septic human PMVEC barrier dysfunction/leak and increased septic PMVEC caspase activation but did not induce any greater degree of septic PMVEC apoptosis compared to isolated PMVEC alone. To date, there have been no studies specifically using human PMVEC in the presence of PMN to assess the connection between septic PMVEC apoptosis and barrier dysfunction. Thus, our comprehensive examination of human septic PMVEC barrier dysfunction in isolated PMVEC and in PMVEC cultured in the presence of PMN clearly establishes for the first time the contributions of apoptosis vs. caspase activation. Collectively, our study suggests that early septic cytomix-induced human PMVEC barrier dysfunction in the presence of PMN is caspase-dependent but is not mediated through PMVEC apoptosis.

The contribution of apoptosis to septic injury and dysfunction of isolated PMVEC *in vitro* has been uncertain. Specifically, there are conflicting data, based on studies examining barrier function in EC from many species and often EC types that are genotypically and phenotypically very distinct from PMVEC and thus of questionable direct relevance to septic lung biology; these include macrovascular pulmonary artery endothelial cells (PAEC) and EC from systemic vascular beds, including macrovascular (e.g., HUVEC) and microvascular (e.g., brain MVEC) (27–31). Most relevant to the present study, EC from different vascular beds exhibit markedly heterogeneous responses to inflammatory environments, especially with respect to the association of apoptosis with increased trans-EC permeability (26–31). Moreover, many studies of EC apoptosis inadequately characterize apoptosis using only one method (e.g., caspase activation or TUNEL) (23, 24, 26).

Death of PMVEC, possibly *via* apoptosis, has been presumed to play a central role in human ARDS, both in the setting of systemic disease like sepsis, but also in direct lung injury (e.g., pneumonia, acid aspiration) (49). There is more evidence to support PMVEC death and specifically apoptosis in various animal models of sepsis and resulting lung injury. We recently reported that PMVEC apoptosis appears to contribute to PMVEC injury and dysfunction in murine cecal ligation/perforation (CLP)-induced sepsis *in vivo* (9, 25). For example, septic pulmonary microvascular barrier dysfunction *in vivo* was temporally associated with and highly correlated with enhanced PMVEC apoptosis, and moreover, inhibition of apoptosis *in vivo* following treatment with Q-VD (a synthetic pan-caspase inhibitor), significantly reduced septic pulmonary microvascular permeability (9, 25). Studies in murine CLP-sepsis by other groups have also demonstrated that inhibition of apoptosis through treatment with siRNA against caspases or FAS-associated death domain rescues septic EC dysfunction, including reducing septic hyper-permeability (46, 50, 51).

Other sub-types of cell death, such as pyroptosis or necrosis, may also be involved in the septic PMVEC barrier dysfunction. Importantly, each cell death sub-type is associated with a specific set of molecular markers (23, 24). For example, both necrosis and necroptosis are thought to be caspase-independent and associated with a loss of cell membrane integrity (i.e., necrotic cells stain positive with PI) (23, 24). Our observation that the increased in PMVEC barrier dysfunction under septic conditions is dependent on caspase activity as well as the apparent lack of any changes in the percentage of PI+ PMVEC under septic conditions or following treatment with Q-VD suggests that necrosis and necroptosis are likely not involved in septic PMVEC barrier dysfunction (23, 24). Pyroptosis is known to be dependent on caspase 1 activity and is also thought to be associated with the formation of pores in the cell membrane (23, 24). Our data found a lack of any significant effect of caspase 1 inhibition on cytomix-stimulated PMVEC barrier dysfunction as well as a lack of changes in the percentage of Annexin V−/PI+ PMVEC under any conditions. Together, this suggests that PMVEC are not undergoing pyroptosis in our model of septic PMVEC barrier dysfunction.

Measurements of TEER and macromolecular permeability are known to reflect different aspects of EC barrier function, and not surprisingly, may be differentially regulated under various inflammatory conditions resulting in barrier dysfunction (52). Our data suggests that while apoptosis occurs in isolated human PMVEC under septic conditions *in vitro* and may be associated with cytomix-induced trans-PMVEC macromolecular flux, it does not appear to be associated with changes in TEER. For example, acute cytomix-induced PMVEC barrier dysfunction was consistent between TEER and both FITC-dextran and EB-albumin techniques. However, this acute septic cytokine-induced PMVEC barrier dysfunction was differentially affected by Q-VD pan-caspase inhibition, which rescued the increased septic macromolecular flux but not septic decreases in TEER. Thus, acute septic trans-PMVEC macromolecular flux appears to be caspase-dependent but septic changes in TEER are not, which is supported by our previous studies using mouse PMVEC (26). Other groups have also reported divergent TEER and macromolecule-leak measurements in studies with PAEC and corneal EC, and have suggested that TEER is inadequately sensitive to biologically important changes in EC barrier function (30, 31). Moreover, the most important feature of PMVEC barrier dysfunction clinically is the increased pulmonary leak of plasma macromolecules, especially albumin accompanied by fluid and resulting in the typical lung edema that characterizes the severe often refractory hypoxemic respiratory failure of septic ARDS (53, 54). The measurement of EB-BSA flux specifically assesses trans-PMVEC permeability to albumin, and is a marker of both paracellular and transcellular permeability pathways (55).

Caspases are multi-functional proteases and while most studies focus on their function in apoptosis, there is clear literature evidence supporting their function in several fundamental cellular processes other than apoptosis (56). Of relevance to this study, multiple caspases have been linked to cleavage and subsequent degradation of the adherens junction adaptor protein β-catenin, including caspases 3, 6, and 8 (57). Furthermore, caspase 3 has also been found to cleave γ-catenin (58). Consequently, it is possible that caspases may regulate PMVEC barrier function through controlling the formation and stability of cell–cell junctions *via* cleavage and processing of critical adapter proteins, such as β-catenin.

PMN have previously been linked to septic PMVEC permeability and this function for PMN was found to be dependent on inducible nitric oxide synthase (iNOS) as the removal of PMN or iNOS deficiency/inhibition restored PMVEC barrier function (9, 32). Thus, it is likely that the mechanism through which PMN mediate PMVEC barrier function is dependent on oxidant/nitrosative stress. Oxidant stress has previously been linked to the activation of caspases, although this function has generally been assumed to lead to apoptosis. Specifically, oxidant stress leads to serial activation of BAX/BAK, mitochondria, and subsequently caspase 9 (59). Moreover, our current data that caspase 9 inhibition in PMVEC cocultured with PMN significantly attenuated cytomix-stimulated permeability but had no effect in PMVEC cultured alone supports this potential function for PMN-induced oxidant stress. Oxidant stress also activates calpains, a family of Ca^2+^-dependent, cytoplasmic cysteine proteases (60). Among several calpain isoforms, the most important and ubiquitously expressed in murine and human cells is calpain1 (μ-calpain). Calpains can cleave many peptide targets, including caspase 3 (60). Thus, it is very plausible that PMN-dependent oxidant stress may result in increased caspase activation and loss of PMVEC barrier function.

Another putative mechanism of microvascular barrier dysfunction is through disruption of MVEC-–extracellular matrix (ECM) interactions resulting in increased MVEC detachment (61). For example, LPS-induced PAEC detachment has been found to be associated with caspase-dependent cleavage of α- and β-catenin as well as focal adhesion kinase, highlighting the ability of caspases to regulate not just inter-EC junctions, but also EC–ECM interactions (62, 63). Interestingly, while caspase inhibition prevented degradation of proteins involved in EC–ECM interactions and rescued EC detachment, it did not prevent LPS-induced disruption of inter-EC junctions or rescue LPS-induced leak (62).

It is likely that there are multiple mechanisms mediating septic EC barrier dysfunction, depending on EC species and type, septic conditions, and timing. For example, stimulation of macrovascular PAEC with a single cytokine (TNFα) resulted in apoptosis as early as 4 h post-stimulation that persisted at 20 h (30). While this apoptosis was also associated with increased permeability across the PAEC monolayer, treatment with Z-VAD did not rescue the enhanced permeability at any time point (30). Furthermore, TNFα has been found to drive loss of corneal EC barrier function through activation of p38 mitogen-activated protein kinase and subsequent disassembly of microtubules, as well as adherens and tight junctions (31). In addition, examination of barrier function in mouse renal MVEC following stimulation with TNFα demonstrated that increased permeability to albumin was associated with altered actin cytoskeleton, as well as formation of gaps between previously confluent cells and a loss of tight junctions and the EC glycocalyx (64). In both of these studies, inhibition of caspase activity (i.e., treatment with caspase inhibitors) had no effect on EC barrier dysfunction (62, 64).

We recognize that our study has limitations. For example, our *in vitro* model of human septic ARDS employed PMVEC cultured alone or with PMN vs. the many different cell types normally present in the lung *in vivo*, such as pericytes and multiple types of circulating inflammatory cells, which interact with PMVEC. Moreover, PMVEC reside *in vivo* on a complete interstitial ECM, and there is an extensive glycocalyx on the surface of the PMVEC, all of which are missing or limited in the *in vitro* setting (65–69). Furthermore, our *in vitro* model employed stimulation with a mixture of three sepsis-relevant cytokines, which is still a less robust septic stimulus than EC would face *in vivo* (e.g., bacterial products such as LPS), as well as the potentially injurious effects of shear stresses associated with blood flow *in vivo*. However, use of this simplified *in vitro* model, as well as the comprehensive assessment of apoptosis (use of three different markers) and PMVEC permeability (use of three complementary measures) allowed for the examination of the function of specifically PMVEC over a comprehensive time course, and thereby the identification of potentially novel mechanisms mediating septic human PMVEC barrier dysfunction.

In addition, the coculture model means that inhibitors, e.g., Q-VD, may have a direct effect on PMN. However, while caspase inhibition has been found to inhibit neutrophil apoptosis and promote neutrophil survival (70), it should be noted that PMN were not exposed to inhibitors (e.g., Q-VD) for the first 3 h of incubation, but only for the final 2 h in cocultures. Moreover, prolonged PMN survival has been shown to increase EC injury and barrier dysfunction (71–73), which was not what we observed in Q-VD treated PMN–PMVEC cocultures. We also recognize that the small *n* = 3 we used to perform our linear correlation analysis could be considered a limitation as it challenges assumptions of normality and homoscedascity. However, we believe our correlation analysis is clearly valid, as it is solidly supported in the literature to use such small *n*’s in order to calculate “*p*” values (74).

In conclusion, our current data suggest for the first time using human PMVEC cultured in the presence or absence of PMN that septic PMVEC barrier dysfunction in the absence of PMN is dependent on apoptosis and caspase activity. However, septic PMVEC barrier dysfunction in the presence of PMN appears to be independent of apoptosis, but very much still caspase-dependent. Future work will pursue the mechanisms of caspase-dependent human PMVEC barrier dysfunction in order to identify potential new therapeutic targets to prevent and treat human septic PMVEC dysfunction in ARDS, and potentially other systemic septic organ dysfunction.

## Ethics Statement

This study was carried out in accordance with the recommendations of the Western University Health Sciences Research Ethics Board with written informed consent from all healthy blood donors. All healthy blood volunteers gave written informed consent in accordance with the Declaration of Helsinki. Discarded lung tissue samples were obtained from the pathology department following removal of all clinical identifiers, and as such, the institutional review board waived the need for written informed consent from lung tissue donors. The protocol was approved by the Western University Health Sciences Research Ethics Board (Protocol #10536E).

## Author Contributions

LW, YA, SW, and MP performed experiments; LW, SM, YA, SW, MP, and SG analyzed data, interpreted results of experiments, and approved final version of manuscript; LW and SG prepared figures; LW, SM, and SG drafted, edited, and revised manuscript; SM and SG conception and design of research.

## Conflict of Interest Statement

The authors declare that the research was conducted in the absence of any commercial or financial relationships that could be construed as a potential conflict of interest.

## Supplementary Material

Figure S1 Schematic of neutrophil (PMN)–pulmonary microvascular endothelial cell (PMVEC) coculture and septic stimulation. PMN and PMVEC were individually stimulated with cytomix (equimolar human tumor necrosis factor α, interleukin 1β, and interferon γ in PBS) at 0.3 and 3 ng/mL vs. PBS control. After 3 h, PMN were added to the corresponding PMVEC monolayer (PMN:PMVEC ratio = 10:1), and cocultured together with or without cytomix stimulation for another 2 h. EC barrier function was assessed by measuring transendothelial electrical resistance (TEER) at 0, 3, and 5 h. To examine trans-PMVEC macromolecular flux, Evans blue (EB)-labeled bovine serum albumin (BSA; 67 kDa) and fluoroisothiocyanate (FITC)-labeled dextran (4 kDa) were added to the apical aspect of the PMVEC (upper chamber of the transwell) at 5 h post-cytomix. The transwell insert was removed at 6 h post-cytomix and the amount of EB-BSA and FITC-dextran in the lower chamber assessed. Note: for the sake of simplicity, the term EC refers specifically to PMVEC within all figures.

Figure S2 Cytomix treatment of human PMVEC induces caspase-dependent intercellular gap formation. **(A)** Cytomix-treated isolated PMVEC had obvious intercellular gaps (arrows) at 5 h vs. PBS, and the gap area in each image was quantified as a percentage of the total PMVEC monolayer area, in both isolated PMVEC [**(B)** EC alone] and for PMN–PMVEC coculture [**(C)** EC + PMN]. The cytomix-induced increase in PMVEC intercellular gaps was completely prevented by pan-caspase inhibition in PMVEC pre-treated with Q-VD **(B,C)**. *N* = 3–4/group. ***p* < 0.01 for cytomix vs. respective PBS; ^##^*p* < 0.01 for Q-VD vs. cytomix alone.
