# Supplementary figures and images for: Differential Mechanisms of Septic Human Pulmonary Microvascular Endothelial Cell Barrier Dysfunction Depending on the Presence of Neutrophils
Source: Front Immunol. 2018 Aug 2;9:1743. doi: 10.3389/fimmu.2018.01743 (PMC6082932; doi:10.3389/fimmu.2018.01743)

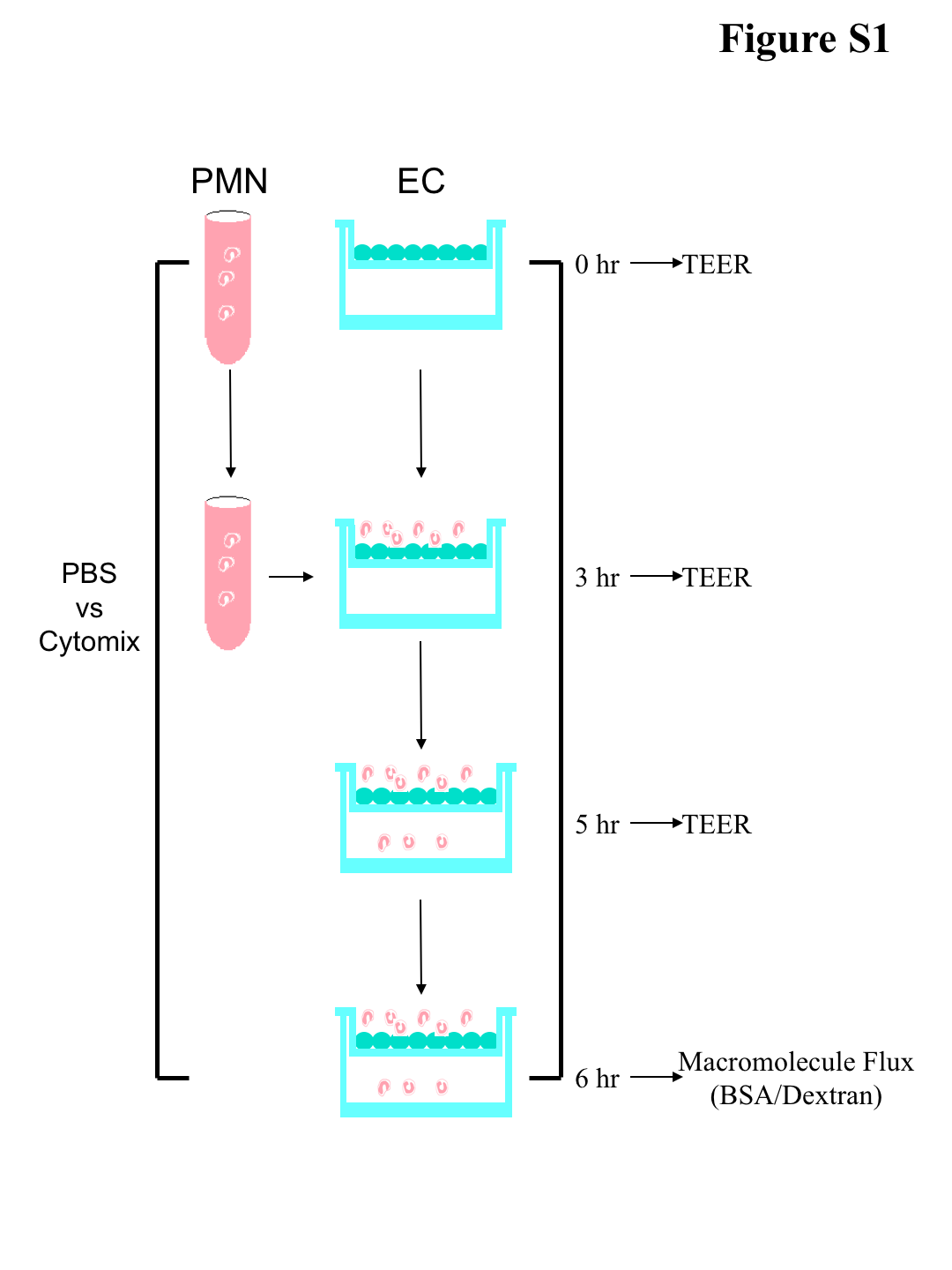

Supplement: Figure S1 — Schematic of neutrophil (PMN)–pulmonary microvascular endothelial cell (PMVEC) coculture and septic stimulation. PMN and PMVEC were individually stimulated with cytomix (equimolar human tumor necrosis factor α, interleukin 1β, and interferon γ in PBS) at 0.3 and 3 ng/mL vs. PBS control. After 3 h, PMN were added to the corresponding PMVEC monolayer (PMN:PMVEC ratio = 10:1), and cocultured together with or without cytomix stimulation for another 2 h. EC barrier function was assessed by measuring transendothelial electrical resistance (TEER) at 0, 3, and 5 h. To examine trans-PMVEC macromolecular flux, Evans blue (EB)-labeled bovine serum albumin (BSA; 67 kDa) and fluoroisothiocyanate (FITC)-labeled dextran (4 kDa) were added to the apical aspect of the PMVEC (upper chamber of the transwell) at 5 h post-cytomix. The transwell insert was removed at 6 h post-cytomix and the amount of EB-BSA and FITC-dextran in the lower chamber assessed. Note: for the sake of simplicity, the term EC refers specifically to PMVEC within all figures. [file image_1.tiff]

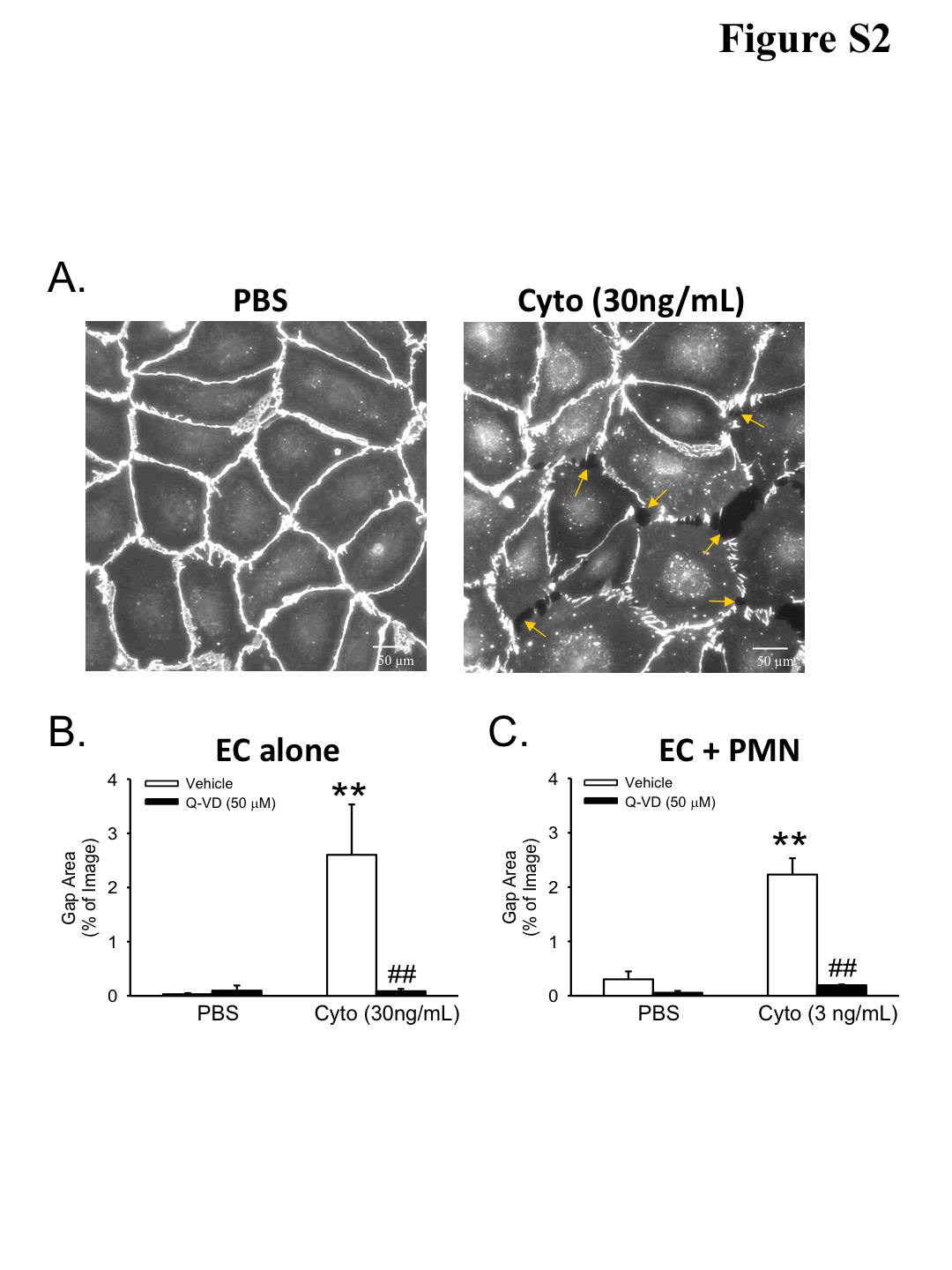

Supplement: Figure S2 — Cytomix treatment of human PMVEC induces caspase-dependent intercellular gap formation. (A) Cytomix-treated isolated PMVEC had obvious intercellular gaps (arrows) at 5 h vs. PBS, and the gap area in each image was quantified as a percentage of the total PMVEC monolayer area, in both isolated PMVEC [(B) EC alone] and for PMN–PMVEC coculture [(C) EC + PMN]. The cytomix-induced increase in PMVEC intercellular gaps was completely prevented by pan-caspase inhibition in PMVEC pre-treated with Q-VD (B,C). N = 3–4/group. **p < 0.01 for cytomix vs. respective PBS; ##p < 0.01 for Q-VD vs. cytomix alone. [file image_2.tiff]
