# Supplementary material for: Differential Mechanisms of Septic Human Pulmonary Microvascular Endothelial Cell Barrier Dysfunction Depending on the Presence of Neutrophils
Source: Front Immunol. 2018 Aug 2;9:1743. doi: 10.3389/fimmu.2018.01743 (PMC6082932; doi:10.3389/fimmu.2018.01743)
Supplement: Supplementary file 3 [file data_sheet_1.PDF]

SUPPLEMENTARY MATERIAL

Differential Mechanisms of Septic Human Pulmonary Microvascular Endothelial Cell Barrier Dysfunction Depending on the Presence of Neutrophils

Lefeng Wang[1,3], Sanjay Mehta[1,2,3], Yousuf Ahmed[1,3], Shelby Wallace[4], M. Cynthia Pape[1,3], and Sean E. Gill[1,2,3,4]

[1]Centre for Critical Illness Research, Lawson Health Research Institute, London, ON, Canada

[2]Division of Respirology, Western University, London, ON, Canada

[3]Department of Medicine, Western University, London, ON, Canada

[4]Department of Physiology and Pharmacology, Western University, London, ON, Canada

Corresponding Author:
Dr. Sean E. Gill
sgill8@uwo.ca

Keywords: human sepsis, endothelial barrier dysfunction, neutrophil, caspase activity

Supplementary: Materials and Methods

Reagents: Goat anti-human vascular endothelial (VE)-cadherin antibody: Santa Cruz Biotechnologies (Mississauga, ON); Donkey anti-goat IgG conjugated to Alexa Fluor 594: Thermo Fisher Scientific (Burlington, ON).

Assessment of PMVEC monolayer intercellular gaps: PMVEC cultured on 1% gelatin-coated 48 well plate to confluence were treated with or without cytomix for 5 hr in the absence (EC alone) or presence of PMN (EC+PMN). The medium was removed and immediately fixed by 100% methanol without washing to avoid cell loss. VE-cadherin staining was carried out as described previously {Arpino:2016gb}. Gap area was calculated as a percentage of the area of each image (200X) using Image J software (National Institutes of Health). Data represent mean of 9-12 images per condition.
